# Longitudinal changes in device-measured physical activity from childhood to young adulthood: the PANCS follow-up study

**DOI:** 10.1186/s12966-024-01578-7

**Published:** 2024-03-06

**Authors:** Anders Husøy, E. Kolle, J. Steene-Johannessen, K.E. Dalene, L. B. Andersen, U. Ekelund, S. A. Anderssen

**Affiliations:** 1 Department of Sports Medicine, Norwegian School of Sport Sciences, Institutt for idrettsmedisinske fag, Norges idrettshøgskole, Pb 4014 Ullevål Stadion, 0806 Oslo, Norway; 2 Department of Chronic Diseases, Norwegian Institute of Public Health, Oslo, Norway; 3 Department of Sport, Food and Natural Sciences, Western Norway University of Applied Sciences, Sogndal, Norway

**Keywords:** Physical activity, Accelerometers, Childhood, Adolescence, Young adulthood

## Abstract

**Background:**

There is a lack of longitudinal studies examining changes in device-measured physical activity and sedentary time from childhood to young adulthood. We aimed to assess changes in device-measured physical activity and sedentary time from childhood, through adolescence, into young adulthood in a Norwegian sample of ostensibly healthy men and women.

**Methods:**

A longitudinal cohort of 731 Norwegian boys and girls (49% girls) participated at age 9 years (2005–2006) and 15 years (2011–2012), and 258 of these participated again at age 24 years (2019–2021; including the COVID-19 pandemic period). Physical activity and sedentary time were measured using ActiGraph accelerometers. Linear mixed models were used to analyse changes in physical activity and sedentary time and whether low levels of childhood physical activity track, i.e., persist into young adulthood (n_change_=721; n_tracking_=640).

**Results:**

The most prominent change occurred between the ages of 9 to 15 years, with an increase in sedentary time (150 min/day) and less time spent in light (125 min/day), moderate (16 min/day), and vigorous physical activity (8 min/day). Only smaller changes were observed between the ages of 15 and 24 years. Changes in moderate-to-vigorous physical activity from childhood to young adulthood differed between subgroups of sex, tertiles of body mass index at baseline and tertiles of peak oxygen uptake at baseline. While the tracking models indicated low absolute stability of physical activity from childhood to young adulthood, children in the lowest quartiles of moderate-to-vigorous (OR:1.88; 95%CI: 1.23, 2.86) and total physical activity (OR: 1.87; 95%CI: 1.21, 2.87) at age 9 years were almost 90% more likely to be in these quartiles at age 24 years compared to those belonging to the upper three quartiles at baseline.

**Conclusions:**

We found a substantial reduction in physical activity and increase in time spent sedentary between age 9 and 15 years. Contrary to previous studies, using mainly self-reported physical activity, little change was observed between adolescence and young adulthood. The least active children were more likely to remain the least active adults and could be targeted for early intervention.

**Supplementary Information:**

The online version contains supplementary material available at 10.1186/s12966-024-01578-7.

## Introduction

Physical activity levels decrease and sedentary time increase from childhood to adolescence, and a large proportion of adolescents does not meet the global physical activity recommendations of 60 min of moderate-to-vigorous physical activity per day [1–3]. The decline in physical activity appears to continue from adolescence into young adulthood [4], and an increase in sedentary behaviour may have serious consequences for population health if persisting into middle- and old-age [5, 6].

The transition from adolescence to young adulthood often involves major life changes, including relocation, higher education, employment, economic independence, relationships, and parenthood. This transitional stage has earlier been associated with a sharp decline in self-reported physical activity [7–10]. Tracking of physical activity from childhood into adulthood, i.e., whether the least active children remain the least active adults, may also be present [11, 12]. Previous studies on tracking of physical activity from childhood to young adulthood have mainly used self-reported physical activity and shown large differences in terms of tracking magnitude, with stability coefficients ranging from − 0.07 to 0.64 [13–24].

There is a paucity of longitudinal studies utilizing device-based measures when examining physical activity and sedentary time during the transition from childhood to young adulthood, and a call has been made for more prospective studies– especially of older adolescents and young adults to get a deeper understanding of physical activity behaviour through major life transitions [1, 4]. Therefore, we aim to describe changes in device-measured physical activity from childhood, through adolescence, into young adulthood in a Norwegian sample of men and women participating in the longitudinal arm of the Physical Activity among Norwegian Children Study (PANCS) [2, 25, 26].

## Materials and methods

### Study sample

In the first Physical Activity among Norwegian Children Study (PANCS1), conducted in 2005–2006, Statistics Norway randomly selected schools across Norway to create a nationally representative sample of 9- and 15-year-olds. In PANCS2 (2011–2012), all participants aged 9 years in PANCS1 were invited to participate again as 15-years-olds. This constituted a longitudinal cohort of 9- and 15-year-olds distributed across 40 schools covering the different geographical regions of Norway. In 2019, all participants from this longitudinal cohort still living in Norway were deemed eligible for inclusion in the PANCS follow-up study. The eligible participants for the PANCS follow-up study were sent an invitation to their registered postal address followed up with a telephone call and complimentary text message a few days later to make sure they had received the invitation. Additionally, we reached out through social media to the eligible participants we could identify from their profiles. Due to the ongoing COVID-19 pandemic, we strived to be flexible in terms of date and time of the data collection while ensuring that the strict hygiene regulations were followed. A hypothesis of 20% reduction in physical activity level from adolescence to young adulthood was formed, with power calculations showing that 182 and 242 participants would be needed in the PANCS follow-up study to achieve 80% and 90% statistical power, respectively.

### Anthropometrics and demographics

Weight and height were measured to the nearest 0.1 kg (Seca 770 and 877, SECA GmbH, Hamburg, Germany) and 0.1 cm (wall-mounted measuring tape), respectively. In PANCS1, participants wore underwear during the anthropometric measurements whereas in PANCS2 and the PANCS follow-up, participants wore light clothing (gym shorts/pants and T-shirt). Therefore, 0.3 kg was subtracted from the bodyweight measures at age 15 and 24 to account for clothing. Body mass index (BMI) was calculated using the standard formula (kg/m^2^). Waist circumference was measured with a tape measure, equally distanced between the edge of the hip and the lower rib. The measure was taken right after exhalation, and to the nearest 0.1 cm. Statistics Norway provided information about parental income and country of birth in PANCS2 (2011–2012). Total parental income was used as a proxy measure for socioeconomic status (SES). Based on the total income of both parents, we categorised the participants into three SES groups: low (total parental income < 750 000 NOK), middle (total parental income 750 000–1 499 999 NOK), and high (total parental income ≥ 1 500 000 NOK). Parents’ country of birth was categorised as number of parents born in Norway (none, one, two).

### Outcome assessment

Physical activity was measured using hip-worn ActiGraph accelerometers (ActiGraph, Pensacola, FL, USA). In PANCS1, we used model CSA7164, in PANCS2 models GT1M and GT3X+, and in PANCS follow-up models GT3X + and GT3X + BT. Participants were instructed to wear the monitor during all waking hours, only removing it for sleep and water-based activities, for a full week in PANCS2 and PANCS follow-up, and for 4 consecutive days (including 2 weekend days) in PANCS1 due to limited monitor storage capacity of the CSA7164.

The accelerometer data were summarised in 10 s epochs, excluding non-wear periods (defined as consecutive spells of zero counts lasting ≥ 60 min) [27]. Data between 00:00 and 06:00 were excluded at age 9 and 15, but not at age 24 due to larger uncertainty surrounding sleeping habits and known nightshift work for several of the participants. A valid day was defined as ≥ 480 min of wear time according to suggested settings for accelerometer data reduction in the International Children’s Accelerometry Database (ICAD) [28], and participants with at least two valid days were included in the analyses [29]. Time spent in intensities ≤ 100 counts per minute (CPM) was classified as sedentary, 101–2295 CPM was classified as light physical activity (LPA), 2296–4011 CPM was classified as moderate physical activity (MPA), and ≥ 4012 CPM was classified as vigorous physical activity (VPA) [30]. All pre- and post-processing of raw accelerometer data were done in ActiLife v6.13.4.

### Aerobic fitness

Peak oxygen uptake (VO_2peak_) was assessed at age 9 years through a maximum exercise test on an electronically braked cycle ergometer (Ergomedic 839E, Monark, Varberg, Sweden). Oxygen uptake was measured every 10 s during the last minutes of the test using a portable MetaMax III X oxygen analyser (Cortex Biophysics, Leipzig, Germany), and the mean of the three highest consecutive measurements was used as VO_2peak_. At age 24 years, VO_2peak_ was measured using a modified Balke protocol on treadmill (Woodway, Weil am Rhein, Germany). Oxygen uptake was registered continuously with 30 s sampling (Oxycon Pro, Jaeger, Würtsburg, Germany), where the highest value was used. Details about these protocols have been described elsewhere [25, 31].

### Other covariates

The geographical area where the participants grew up was categorised into four distinctive regions of Norway (Central Norway, East, Southwest, and North), and was determined from the location of the participants primary schools.

Pubertal status was assessed at age 9 by trained personnel according to the 5-point Tanner classification system [32]. The assessment score of genitalia and the assessment score of breasts were used for boys and girls respectively, while the score for pubic hair was used if one of the formers was missing. Due to little variation and low scores on the Tanner scale, the variable was reclassified into prepubertal (Tanner score = 1) and started puberty (Tanner score > 1).

### Statistical analyses

All analyses were performed in R v4.2.0 [33]. A linear mixed model was used to analyse both change and tracking of physical activity over time. All models included a nested random intercept at an individual level within schools at age 9 years. If model singularity became an issue, the complexity of the random factors was reduced to a random intercept at school level at age 9 years. Missing outcome data were handled by the restricted maximum likelihood estimator in the mixed models, which includes all available data (n_change_=721; n_tracking_=640) under the missing at random assumption, i.e., missing data can be explained by the observed data [34].

The analyses of change in sedentary time and physical activity were modelled with time itself as the main effect of interest and sedentary time and the different physical activity intensities as outcomes. Adjustments were made for age, sex, wear time of the accelerometer, wear month, and a weekday/weekend wear day ratio (number of valid weekdays divided by number of valid weekend days). Interaction terms between time and sex, time and VO_2peak_ tertiles at baseline, time and BMI tertiles at baseline, and time and parental income were sequentially added to the model to assess whether change in moderate-to-vigorous physical activity (MVPA) differed between these subgroups.

The analyses of tracking were modelled as the initial measurement of MVPA (minutes per day) and total physical activity (counts per minute) regressed on the subsequent values of the same measurement. Physical activity variables at each time point were standardised as z-scores adjusted for wear time, wear month and a weekday/weekend ratio. All models were adjusted for sex, parental income, parents’ country of birth, geographical region, pubertal status, follow-up time and baseline values of age, body mass index and maximal oxygen uptake. Crude models, only modelling the initial physical activity measurement regressed on the subsequent measures, were also included to display the effect of the adjustments. Tracking strength was evaluated with stability coefficients, which could take on values between − 1 and 1 and interpreted as longitudinal correlation coefficients. Stability coefficients < 0.30 were considered weak tracking, 0.30 to 0.60 moderate tracking, and > 0.60 strong tracking [35]. We also assessed whether belonging to either the lowest or highest quartile of MVPA or total physical activity at age 9 years could predict belonging to the same quartile later in life. Consequently, MVPA and total physical activity were divided into quartiles, and odds ratios were calculated for participants in the lowest and highest quartiles (compared to participants belonging to any of the other three quartiles).

## Results

**Fig. 1:**
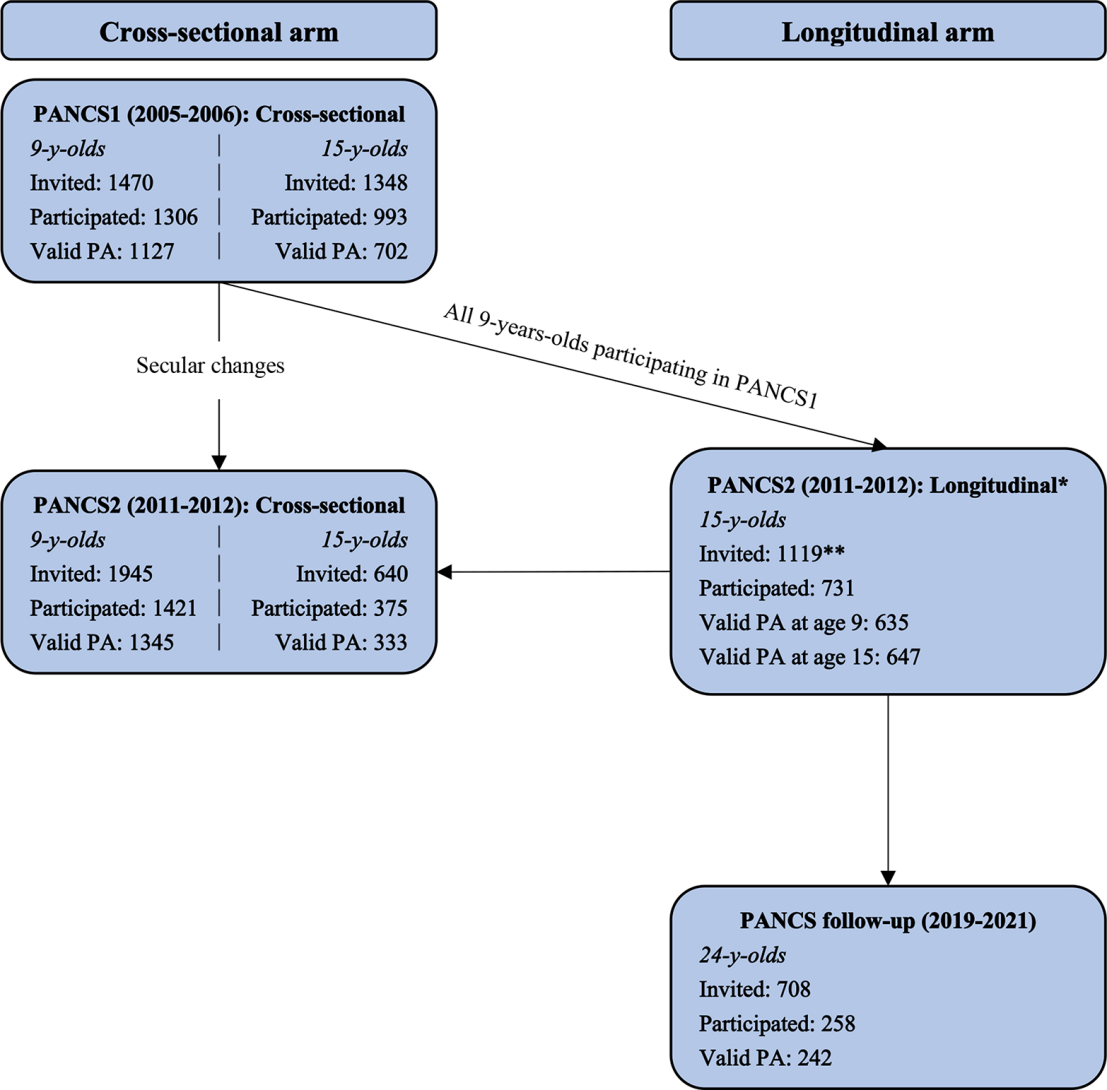
Flow chart of the inclusion process in the cross-sectional and longitudinal study arms of PANCS. PA: Physical activity. *Also included in the cross-sectional part of PANCS2. **1119 of the 1306 9-years-olds who participated in PANCS1 were identified and invited to participate again in PANCS2 as 15-year-olds

In PANCS2 (2011–2012), 731 boys and girls who had participated in PANCS1 as 9-year-olds (2006–2006) participated again as 15-year-olds (1119 eligible participants). In 2019, all 731 participants still living in Norway (*n* = 708) were deemed eligible for inclusion in the PANCS follow-up study. Of these, 258 were included in the present follow-up. A flow chart of the inclusion process is shown in Fig. 1. The main reason for non-participation in adolescence was no response to the written informed consent form sent to the primary guardians, while a lack of interest and scepticism towards domestic travelling during the COVID-19 pandemic were cited as main reasons not to participate in the follow-up study in young adulthood.

Descriptive statistics at each time point can be found in Table 1. The mean age at the three data collections was 9.6 years in PANCS1, 15.2 years in PANCS2, and 24.6 years in PANCS follow-up– giving an average follow-up length of 14.9 (SD 0.7) years. The percentage of boys and girls participating in PANCS1 and 2 were 51% and 49% respectively. Slightly more young women (56%) than young men opted to participate in PANCS follow-up. The proportion of participants across socioeconomic status remained relatively stable over time, while the proportion of participants with two parents born in Norway were somewhat higher in PANCS follow-up compared to PANCS1 and 2 (78% vs. $\sim$72%). Number of valid days of accelerometer-wear did not differ between age 15 and 24 years but was lower at age 9 years due to the storage limitations of the accelerometers deployed in PANCS1.

**Table 1:** Descriptive statistics at 9, 15 and 24 years of age

Variable	Missing	PANCS1 *n* = 635^*a*^	PANCS2 *n* = 647^*a*^	PANCS follow-up *n* = 242^*a*^
Age (yrs)	21%	9.6 (0.4)	15.2 (0.6)	24.6 (0.8)
Height (cm)	25%	139.0 (6.5)	169.7 (8.4)	174.5 (10.0)
Weight (kg)	25%	33.5 (6.5)	59.8 (10.7)	73.1 (14.3)
Waist Circumference (cm)	27%	61.9 (7.2)	70.9 (7.5)	80.1 (10.4)
BMI (kg/m^2^)	25%	17.2 (2.5)	20.7 (3.0)	23.9 (4.0)
VO_2peak_ (ml/kg/min)	56%	46.4 (7.4)	NA	48.5 (9.9)
Sex	0%			
Female		309 (49%)	318 (49%)	136 (56%)
Male		326 (51%)	329 (51%)	106 (44%)
Pubertal status (2005–2006)	1.4%			
Pre-pubertal		545 (86%)	543 (85%)	210 (87%)
Started puberty		86 (14%)	95 (15%)	32 (13%)
Region (2005–2006)	16%			
Central Norway		24 (5%)	24 (4%)	7 (3%)
East		282 (53%)	286 (52%)	126 (61%)
North		42 (8%)	53 (10%)	21 (10%)
Southwest		179 (34%)	183 (34%)	53 (26%)
Parental Income (2011–2012)	0.1%			
Low		131 (21%)	134 (21%)	40 (17%)
Middle		370 (58%)	378 (58%)	146 (60%)
High		133 (21%)	135 (21%)	56 (23%)
Parents born in Norway	0%			
None		57 (9%)	64 (10%)	14 (6%)
One		119 (19%)	122 (19%)	39 (16%)
Two		459 (72%)	461 (71%)	189 (78%)
*Accelerometry*				
Valid Days	30%	3.7 (0.6)	6.4 (1.9)	7.0 (1.2)
Weekday	30%	1.9 (0.3)	4.8 (1.4)	5.1 (0.9)
Weekend	30%	1.8 (0.5)	1.6 (0.9)	1.9 (0.7)
Wear time (hrs/day)	30%	13.2 (1.1)	13.5 (1.4)	13.8 (1.5)
Sedentary (min/day)	30%	435.3 (70.8)	590.6 (73.3)	596.9 (85.7)
LPA (min/day)	30%	280.9 (47.4)	161.2 (39.6)	178.4 (53.1)
MPA (min/day)	30%	44.9 (14.1)	31.3 (11.5)	27.2 (12.7)
VPA (min/day)	30%	30.6 (16.0)	24.8 (14.8)	26.5 (17.3)
MVPA (min/day)	30%	76 (27)	56 (23)	54 (23)
Total PA (counts/min)	30%	719.5 (251.9)	444.2 (159.3)	435.5 (150.4)

NA: Not available; BMI: Body mass index; VO_2peak_: Peak oxygen uptake; LPA: Light physical activity; MPA: Moderate physical activity; VPA: Vigorous physical activity

^*a*^ Mean (SD); n (%); Number of participants defined by valid accelerometer data at each time point

**Fig. 2:**
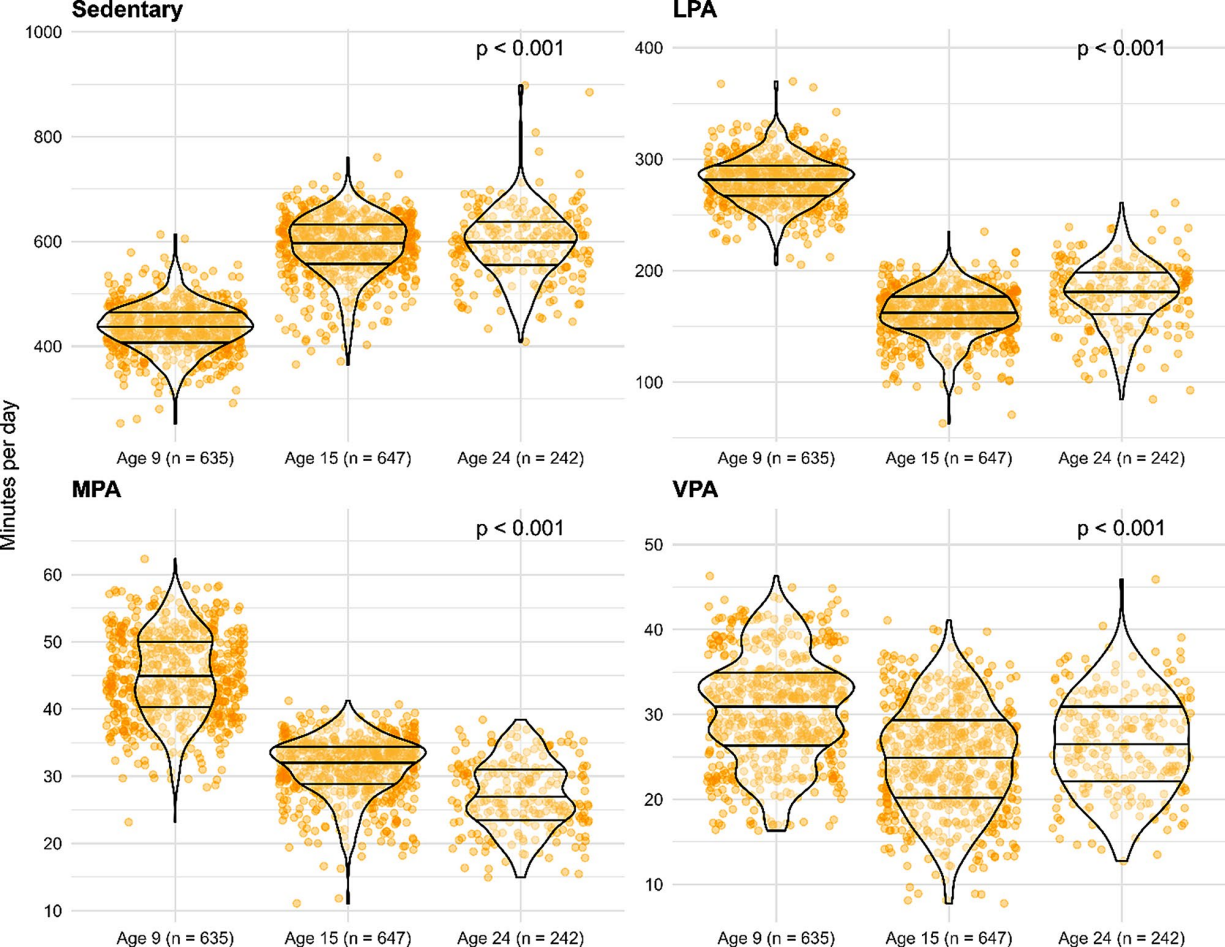
Violin plot of average daily time spent sedentary and in different physical activity intensities from 9 to 24 years of age. Values are adjusted for sex, wear time, weekday to weekend wear ratio, and wear month of the accelerometer. Wider parts of the figures indicate more observations, and narrower parts indicate fewer observations. Quartiles are described by the solid, horizontal lines. LPA: Light physical activity; MPA: Moderate physical activity; VPA: Vigorous physical activity

Substantial changes in physical activity and sedentary time were observed between age 9 years and age 24 years (all *p* < 0.001; *n* = 721). The most prominent change occurred from age 9 to 15 years, with a marked increase in time spent sedentary (150 min/day) combined with less time spent in LPA (125 min/day), MPA (16 min/day) and VPA (8 min/day). We observed smaller changes between 15 and 24 years of age, with an increase in daily time spent in LPA (11 min/day) and VPA (3 min/day)– and a decrease in MPA (4 min/day) and time spent sedentary (10 min/day). Figure 2 visualises the observed data of time spent sedentary and in LPA, MPA, and VPA at all time-points.

**Fig. 3:**
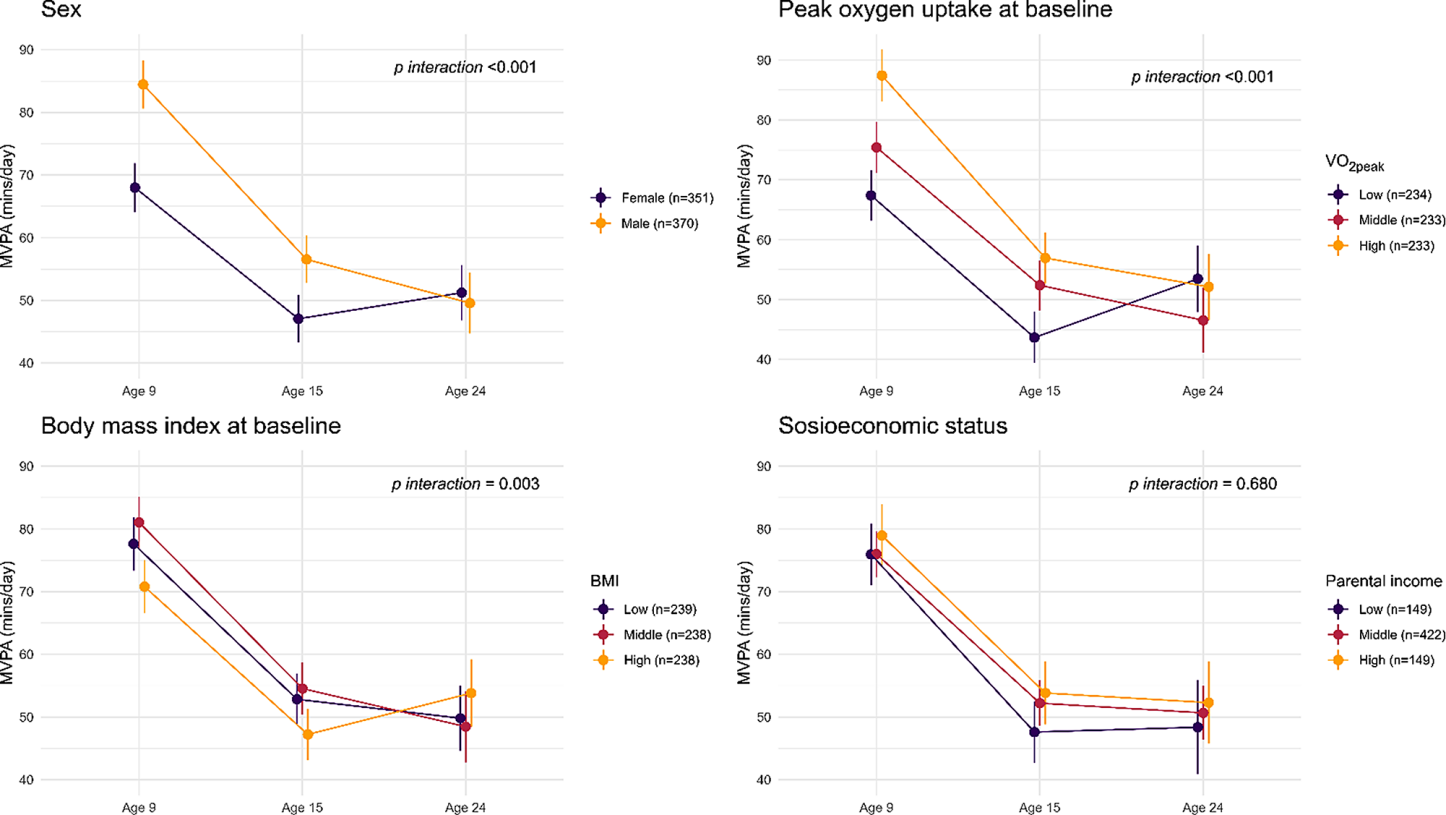
Changes in daily moderate-to-vigorous physical activity (MVPA) from 9 to 24 years of age, by subgroups of sex, VO_2peak_ at baseline, body mass index at baseline, and parental income

Time spent in MVPA decreased notably from 76.3 min/day to 51.6 min/day (Change: −24.7 min/day; 95% CI: −29.1, − 20.4) between the ages of 9 to 15 years. Between the ages of 15 to 24 years, time spent in MVPA remained unchanged (Change: −0.5 min/day; 95% CI: −5.1, 4.3). Several factors modified how the daily amount of MVPA changed over time– such as sex, BMI tertiles at baseline and VO_2peak_ tertiles at baseline (Fig. 3). For example, boys were more active than girls at age 9 and 15 years whereas this difference was eliminated at age 24 years. Further, those with highest BMI and lowest VO_2peak_ were least active at age 9 and 15 years but equally or more active than other groups at age 24 years. The change in MVPA over time did not differ between subgroups of parental income. We did, however, observe a trend where participants with the highest-earning parents consistently had the highest amount of MVPA per day at all time-points.

The stability coefficients indicate weak tracking of physical activity from age 9 to age 24 (Table 2). The odds ratios, however, reveal that those in the lowest quartile of MVPA (Odds ratio: 1.88; 95% CI: 1.23, 2.86) and total physical activity (Odds ratio: 1.87; 95% CI: 1.21, 2.87) at age 9 were almost 90% more likely to be in these quartiles at age 24 as compared to those belonging to any of the upper three quartiles at 9 years of age (*p* = 0.004). Furthermore, the tracking of physical activity was slightly stronger among females than males (not shown), and from age 9 to 15 as compared to age 15 to 24.

**Table 2:** Tracking of device-measured physical activity from 9 to 24 years of age

	Crude models		Adjusted models	
Tracking age	MVPA	Total PA	MVPA	Total PA
*9–24 (n = 640)*	*Estimate (95% CI)*	*Estimate (95% CI)*	*Estimate (95% CI)*	*Estimate (95% CI)*
Stability Coefficient*	0.22 (0.14, 0.29)	0.22 (0.14, 0.29)	0.18 (0.09, 0.26)	0.16 (0.08, 0.24)
Odds Ratio				
Lowest Quartile	1.92 (1.33, 2.76)	2.10 (1.46, 3.01)	1.88 (1.23, 2.86)	1.87 (1.21, 2.87)
Highest Quartile	1.44 (0.99, 2.08)	1.38 (0.97, 1.97)	1.07 (0.69, 1.66)	1.15 (0.75, 1.76)
*9–15 (n = 458)*				
Stability Coefficient*	0.29 (0.21, 0.37)	0.28 (0.20, 0.36)	0.24 (0.15, 0.33)	0.23 (0.13, 0.32)
Odds Ratio				
Lowest Quartile	2.23 (1.47, 3.39)	2.65 (1.75, 4.03)	2.18 (1.34, 3.55)	2.41 (1.47, 3.96)
Highest Quartile	1.83 (1.17, 2.85)	1.67 (1.10, 2.55)	1.23 (0.73, 2.08)	1.40 (0.85, 2.30)
*15–24 (n = 163)*				
Stability Coefficient*	0.00 (− 0.15, 0.15)	0.06 (− 0.09, 0.21)	−0.05 (− 0.22, 0.12)	0.04 (− 0.14, 0.21)
Odds Ratio				
Lowest Quartile	1.54 (0.74, 3.22)	1.72 (0.86, 3.47)	1.78 (0.68, 4.68)	1.92 (0.79, 4.69)
Highest Quartile	0.80 (0.39, 1.67)	1.75 (0.90, 3.41)	0.86 (0.34, 2.16)	1.74 (0.74, 4.08)

MVPA: Moderate and vigorous physical activity; PA: Physical activity

*Weak tracking: <0.30, moderate tracking: 0.30–0.60, strong tracking: >0.60

## Discussion

There was a substantial reduction in physical activity from age 9 to 15 years, and an increase in sedentary time. From the age of 15 to 24 years, these variables remained relatively stable. The change in MVPA over time differed between subgroups of sex, BMI tertiles at baseline and VO_2peak_ tertiles at baseline. The tracking models indicated low absolute stability in MVPA and total physical activity from childhood to young adulthood. However, those in the lowest quartile of total physical activity and MVPA at age 9 years were more likely to be in the lowest quartile at age 24 years compared with those who belonged to any of the upper three quartiles at 9 years.

Our study is unique in terms of study design, methods, and length of follow-up. The most comparable study was carried out by Ortega et al. [3], who investigated change in objectively measured physical activity in two different cohorts covering age groups from childhood to adolescence and from adolescence to young adulthood. They found an average decline in MVPA from childhood to adolescence of $\sim$30 min/day, and a further decline in MVPA from adolescence to early adulthood of $\sim$13 min/day. Additionally, an average increase of 2 h and 45 min of sedentary time per day from childhood to adolescence was reported. While these results are comparable to ours in terms of change from childhood to adolescence, we did not find an overall change from adolescence to early adulthood of the same magnitude. Ortega et al. [3] do, however, emphasise the fact that the study describes changes in two separate age cohorts– one from childhood to adolescence, and one from adolescence to young adulthood. Thus, the results cannot be interpreted as longitudinal changes from childhood to young adulthood. Earlier studies using accelerometer data have shown large discrepancies in change in MVPA from childhood to adolescence, such as a mean decline of $\sim$140 min per day from age 9 to 15 years in a sample of American youth [36] compared to a mean decline of 8.4 min per day over the same time-span in a harmonised review of European studies [37]. The large difference in results may in part be due to differences in methods such as choice of accelerometer cut-points [38], and perhaps also geographical, cultural and temporal differences. Studies on change in device-measured physical activity from adolescence to young adulthood have been scarce, but a recent review found nine studies using accelerometer data that showed a mean decline of 7.4 min of daily MVPA between adolescence and adulthood [4]. When restricting the age in adolescence to ≤ 15 years and age in adulthood to ≥ 18 years, only two studies using accelerometers were identified [4]– highlighting the need for longitudinal studies from adolescence to adulthood using device-measured physical activity.

Several studies have investigated the potential tracking of physical activity from childhood to adulthood, most commonly using self-reported physical activity and rarely adjusting for potential confounding variables– making any direct comparison with our study difficult [13–24]. In these studies, most stability coefficients range between 0.2 and 0.5. The corresponding stability coefficients in our study places in the lower end of that range, indicating low stability of physical activity over time in our Norwegian sample. The predictability of physical activity in young adulthood from early measurements did show potential on a group level, however, as the participants belonging to the lowest quartile of MVPA and total physical activity at age 9 years were substantially more likely to be in that quartile at age 24 years as compared to those belonging to any of the upper three quartiles at 9 years. Not shown in the results, a machine learning algorithm (eXtreme Gradient Boosting) was applied to analyse whether all collected variables at age 9 and 15 years could predict whether a participant belonged to the upper or lower half of total physical activity at age 24 years (Supplementary Material 4, page 1). The algorithm correctly classified a participant’s physical activity level in young adulthood (high/low) 53% of the time (i.e., close to a random guess). This corroborates our somewhat unexpected finding in Fig. 3, where subgroups with the lowest amount of daily MVPA at age 9 years ended up with the highest amount at age 24 years, i.e., girls, the lowest tertile of baseline VO_2peak_, and the highest tertile of baseline BMI. These findings may suggest that the transitional phase between adolescence and adulthood could be formative of physical activity behaviour in young adulthood.

Physical activity was measured using three generations of the Actigraph accelerometer. An earlier comparison of these generations showed that the piezoelectric accelerometer inside the Actigraph model CSA7164 records less sedentary time and higher total activity than the newer models GT1M and GT3X+, used at age 15 and 24 years, based on a Micro-Electro-Mechanical System [39]. When analysing raw data from the Actigraph GT3X+, a low frequency extension filter is available shown to almost completely attenuate the inter-model differences in sedentary time [40]. However, differences may, to a lesser extent, increase at the higher intensities when enabling this filter [40]. As a focus in our study was on how physical activity changed over time, we did not enable this filter. Changes in physical activity– and especially sedentary time– should therefore be interpreted somewhat cautiously. Nevertheless, the beforementioned inter-model differences are relatively small in magnitude and much smaller than the changes we observed between 9 and 15 years. Thus, we do not suspect that measuring physical activity with the same device across all time points would have altered our conclusions.

The strengths of this study include repeated measurements of physical activity using accelerometers in a sample of Norwegian men and women covering all regions of Norway, and the novelty of describing longitudinal changes in device-measured physical activity from childhood to young adulthood– which to our knowledge has not been previously described. While Statistics Norway randomly selected schools for participation in childhood to create a nationally representative sample, no steps were taken at age 24 years to ensure that the sample was representative of young Norwegian adults. However, the follow-up cohort at age 24 years was comparable to other Norwegian studies of young adults in several aspects, such as BMI, maximal oxygen uptake, and total physical activity level [31, 41, 42]. There are some limitations to the current study. The accelerometer data were aggregated in 10 s epochs at age 9 and 15 years, and the raw accelerometer files were not available to change epoch length. Therefore, 10 s epochs were also used at age 24 years to be consisted with previous data. This may have led to an underestimation of physical activity at age 9 years due to children’s tendency of being active intermittently in short bursts [43, 44]. Another weakness is the loss to follow-up from age 15 to 24 years, where 258 out of a potential 708 participants ($\sim$36%) partook in the study. There is, however, no established cut-off as to how much missing data is too much. The observations lost to follow-up are often categorised as either missing completely at random (MCAR), missing at random (MAR) or missing not at random (MNAR) [34]. A simulation study by Kristman et al. [45] found no important bias for the MCAR or the MAR mechanism on any level of missing data (up to 60%), implying that loss to follow-up characterised by these types of missing data can be handled properly in the analysis– such as the restricted maximum likelihood estimator applied in the mixed model. Missing data through the MNAR mechanism showed a different pattern, with serious biased estimates already at a missingness of 20% [45]. A comparison of participants completing all three studies with those who dropped out can be found in Supplementary Material 4 (page 2), revealing some differences in waist circumference, sex, geographical region, socioeconomic status, number of Norwegian parents, and accelerometer wear. The large number of variables in our dataset is supportive of the MAR assumption, and we are confident most of the missing data are explained by the observed data. It should also be noted that most of the data collection in the PANCS follow-up study was carried out during the ongoing COVID-19 pandemic. Many of the invited declined to participate due to restrictions, a high local infection rate in Oslo, and a general recommendation throughout the pandemic to avoid any unnecessary domestic travels. Thus, the participation rate may have been higher if the study had been carried out under normal circumstances.

Future prospective studies using device-measured physical activity are encouraged to cover the transitional phase from adolescence to adulthood, as this may be a critical period in shaping physical activity habits and behaviour in young adulthood. Additionally, the least active children were at higher odds ending up as the least active adults than their more active peers and could be targeted for early intervention.

## Conclusions

Physical activity declined, and time spent sedentary increased from childhood to adolescence. From adolescence to young adulthood, these variables remained relatively stable. Tracking of physical activity from childhood to young adulthood was weak in terms of stability coefficients, but those in the lowest quartile of MVPA and total physical activity at age 9 years were almost 90% more likely to be in that quartile at age 24 years as compared to those belonging to any of the three upper quartiles at baseline.

### Electronic supplementary material

Below is the link to the electronic supplementary material.

Supplementary Material 1

Supplementary Material 2

Supplementary Material 3

Supplementary Material 4

## Data Availability

De-identified individual participant data that underlie the results reported in this Article can be accessed upon reasonable request after publication. Requests should be directed to the corresponding author and needs to be approved by the principal investigators (UE and SAA). Analytical code (R scripts) will be made available upon request.

## Abbreviations

PANCS: Physical Activity among Norwegian Children Study
BMI: Body Mass Index
SES: Socioeconomic Status
CPM: Counts pr minute
LPA: Light physical activity
MPA: Moderate physical activity
VPA: Vigorous physical activity
VO_2peak_: Peak oxygen uptake
MVPA: Moderate-to-vigorous physical activity
MCAR: Missing completely at random
MAR: Missing at random
MNAR: Missing not at random

## References

[1] van Sluijs EMF, Ekelund U, Crochemore-Silva I, Guthold R, Ha A, Lubans D et al. Physical activity behaviours in adolescence: current evidence and opportunities for intervention. Lancet [Internet]. 2021;398:429–42. 10.1016/S0140-6736(21)01259-9. 10.1016/S0140-6736(21)01259-9 PMC7612669 34302767

[2] Dalene KE, Anderssen SA, Andersen LB, Steene-Johannessen J, Ekelund U, Hansen BH et al. Secular and longitudinal physical activity changes in population-based samples of children and adolescents. Scand J Med Sci Sports [Internet]. 2018;28:161–71. Available from: https://onlinelibrary.wiley.com/doi/abs/10.1111/sms.12876. 10.1111/sms.12876 28299832

[3] Ortega FB, Konstabel K, Pasquali E, Ruiz JR, Hurtig-Wennlöf A, Mäestu J et al. Objectively measured physical activity and sedentary time during childhood, adolescence and young adulthood: a cohort study. PLoS One [Internet]. 2013;8:e60871. 10.1371/journal.pone.0060871. 10.1371/journal.pone.0060871 PMC3634054 23637772

[4] Corder K, Winpenny E, Love R, Brown HE, White M, van Sluijs E. Change in physical activity from adolescence to early adulthood: a systematic review and meta-analysis of longitudinal cohort studies. Br J Sports Med [Internet]. 2019;53:496–503. 10.1136/bjsports-2016-097330. 10.1136/bjsports-2016-097330 PMC6250429 28739834

[5] Lee I-M, Shiroma EJ, Lobelo F, Puska P, Blair SN, Katzmarzyk PT et al. Effect of physical inactivity on major non-communicable diseases worldwide: an analysis of burden of disease and life expectancy. Lancet [Internet]. 2012;380:219–29. 10.1016/S0140-6736(12)61031-9. 10.1016/S0140-6736(12)61031-9 PMC3645500 22818936

[6] Katzmarzyk PT, Friedenreich C, Shiroma EJ, Lee I-M. Physical inactivity and non-communicable disease burden in low-income, middle-income and high-income countries. Br J Sports Med [Internet]. 2022;56:101–6. 10.1136/bjsports-2020-103640. 10.1136/bjsports-2020-103640 PMC8478970 33782046

[7] Baranowski T, Cullen KW, Basen-Engquist K, Wetter DW, Cummings S, Martineau DS et al. Transitions out of high school: time of increased cancer risk? Prev Med [Internet]. 1997;26:694–703. 10.1006/pmed.1997.0193. 10.1006/pmed.1997.0193 9327479

[8] Caspersen CJ, Pereira MA, Curran KM. Changes in physical activity patterns in the United States, by sex and cross-sectional age. Med Sci Sports Exerc [Internet]. 2000;32:1601–9. 10.1097/00005768-200009000-00013. 10.1097/00005768-200009000-00013 10994912

[9] Leslie E, Fotheringham MJ, Owen N, Bauman A. Age-related differences in physical activity levels of young adults. Med Sci Sports Exerc [Internet]. 2001;33:255–8. 10.1097/00005768-200102000-00014. 10.1097/00005768-200102000-00014 11224815

[10] Leslie E, Owen N, Salmon J, Bauman A, Sallis JF, Lo SK. Insufficiently active Australian college students: perceived personal, social, and environmental influences. Prev Med [Internet]. 1999;28:20–7. 10.1006/pmed.1998.0375. 10.1006/pmed.1998.0375 9973584

[11] Hayes G, Dowd KP, MacDonncha C, Donnelly AE. Tracking of Physical Activity and Sedentary Behavior From Adolescence to Young Adulthood: A Systematic Literature Review. J Adolesc Health [Internet]. 2019;65:446–54. 10.1016/j.jadohealth.2019.03.013. 10.1016/j.jadohealth.2019.03.013 31248803

[12] Telama R. Tracking of physical activity from childhood to adulthood: a review. Obes Facts [Internet]. 2009;2:187–95. 10.1159/000222244. 10.1159/000222244 PMC6516203 20054224

[13] Aarnio M, Winter T, Peltonen J, Kujala UM, Kaprio J. Stability of leisure-time physical activity during adolescence–a longitudinal study among 16-, 17- and 18-year-old Finnish youth. Scand J Med Sci Sports [Internet]. 2002;12:179–85. 10.1034/j.1600-0838.2002.00250.x. 10.1034/j.1600-0838.2002.00250.x 12135451

[14] Andersen LB, Haraldsdóttir J. Tracking of cardiovascular disease risk factors including maximal oxygen uptake and physical activity from late teenage to adulthood. An 8-year follow-up study. J Intern Med [Internet]. 1993;234:309–15. 10.1111/j.1365-2796.1993.tb00748.x. 10.1111/j.1365-2796.1993.tb00748.x 8354982

[15] Anderssen N, Wold B, Torsheim T. Tracking of physical activity in adolescence. Res Q Exerc Sport [Internet]. 2005;76:119–29. 10.1080/02701367.2005.10599274. 10.1080/02701367.2005.10599274 16128480

[16] Azevedo MR, Menezes AM, Assunção MC, Gonçalves H, Arumi I, Horta BL et al. Tracking of physical activity during adolescence: the 1993 Pelotas Birth Cohort, Brazil. Rev Saúde Pública [Internet]. 2014 [cited 2021 Oct 4];48:925–30. Available from: https://www.scielo.br/scielo.php?script=sci_arttext&pid=S0034-89102014000600925 10.1590/S0034-8910.2014048005313 PMC4285821 26039395

[17] Boreham C, Robson PJ, Gallagher AM, Cran GW, Savage JM, Murray LJ. Tracking of physical activity, fitness, body composition and diet from adolescence to young adulthood: The Young Hearts Project, Northern Ireland. Int J Behav Nutr Phys Act [Internet]. 2004;1:14. 10.1186/1479-5868-1-14. 10.1186/1479-5868-1-14 PMC524366 15462676

[18] Cleland V, Dwyer T, Venn A. Which domains of childhood physical activity predict physical activity in adulthood? A 20-year prospective tracking study. Br J Sports Med [Internet]. 2012;46:595–602. 10.1136/bjsports-2011-090508. 10.1136/bjsports-2011-090508 22144006

[19] Glenmark B, Hedberg G, Jansson E. Prediction of physical activity level in adulthood by physical characteristics, physical performance and physical activity in adolescence: an 11-year follow-up study. Eur J Appl Physiol Occup Physiol [Internet]. 1994;69:530–8. 10.1007/BF00239871. 10.1007/BF00239871 7713074

[20] Rauner A, Jekauc D, Mess F, Schmidt S, Woll A. Tracking physical activity in different settings from late childhood to early adulthood in Germany: the MoMo longitudinal study. BMC Public Health [Internet]. 2015;15:391. 10.1186/s12889-015-1731-4. 10.1186/s12889-015-1731-4 PMC4407713 25887314

[21] Raustorp A, Fröberg A. Tracking of Pedometer-Determined Physical Activity: A 16-Year Follow-Up Study. J Phys Act Health [Internet]. 2018;15:7–12. 10.1123/jpah.2017-0146. 10.1123/jpah.2017-0146 28771068

[22] Suppli CH, Due P, Henriksen PW, Rayce SLB, Holstein BE, Rasmussen M. Low vigorous physical activity at ages 15, 19 and 27: childhood socio-economic position modifies the tracking pattern. Eur J Public Health [Internet]. 2013;23:19–24. 10.1093/eurpub/cks040. 10.1093/eurpub/cks040 22552259

[23] Telama R, Yang X, Viikari J, Välimäki I, Wanne O, Raitakari O. Physical activity from childhood to adulthood: a 21-year tracking study. Am J Prev Med [Internet]. 2005;28:267–73. 10.1016/j.amepre.2004.12.003. 10.1016/j.amepre.2004.12.003 15766614

[24] Twisk JW, Kemper HC, van Mechelen W. Tracking of activity and fitness and the relationship with cardiovascular disease risk factors. Med Sci Sports Exerc [Internet]. 2000;32:1455–61. 10.1097/00005768-200008000-00014. 10.1097/00005768-200008000-00014 10949012

[25] Kolle E, Steene-Johannessen J, Andersen LB, Anderssen SA. Objectively assessed physical activity and aerobic fitness in a population-based sample of Norwegian 9- and 15-year-olds. Scandinavian Journal of Medicine & Science in Sports [Internet]. 2010;20:e41–7. 10.1111/j.1600-0838.2009.00892.x. 10.1111/j.1600-0838.2009.00892.x 19422647

[26] Steene-Johannessen J, Anderssen SA, Kolle E, Hansen BH, Bratteteig M, Dalhaug EM et al. Temporal trends in physical activity levels across more than a decade - a national physical activity surveillance system among Norwegian children and adolescents. Int J Behav Nutr Phys Act [Internet]. 2021;18:55. 10.1186/s12966-021-01120-z. 10.1186/s12966-021-01120-z PMC8074468 33902618

[27] Aadland E, Andersen LB, Anderssen SA, Resaland GK. A comparison of 10 accelerometer non-wear time criteria and logbooks in children. BMC Public Health [Internet]. 2018;18. 10.1186/s12889-018-5212-4. 10.1186/s12889-018-5212-4 PMC5840816 29510709

[28] International Children’s Accelerometry Database (ICAD) [Internet]. MRC Epidemiology Unit. 2014 [cited 2023 Dec 18]. Available from: https://www.mrc-epid.cam.ac.uk/research/studies/icad/.

[29] Rich C, Geraci M, Griffiths L, Sera F, Dezateux C, Cortina-Borja M. Quality control methods in accelerometer data processing: defining minimum wear time. PLoS One [Internet]. 2013;8:e67206. 10.1371/journal.pone.0067206. 10.1371/journal.pone.0067206 PMC3691227 23826236

[30] Evenson KR, Catellier DJ, Gill K, Ondrak KS, McMurray RG. Calibration of two objective measures of physical activity for children. J Sports Sci [Internet]. 2008;26:1557–65. 10.1080/02640410802334196. 10.1080/02640410802334196 18949660

[31] Edvardsen E, Hansen BH, Holme IM, Dyrstad SM, Anderssen SA. Reference values for cardiorespiratory response and fitness on the treadmill in a 20- to 85-year-old population. Chest [Internet]. 2013;144:241–8. 10.1378/chest.12-1458. 10.1378/chest.12-1458 23287878

[32] Tanner JM. Growth at adolescence: with a general consideration of the effects of Hereditary and Environmental factors upon growth and maturation from birth to Maturity. Blackwell Scientific; 1962.

[33] The R Project for Statistical Computing. [Internet]. [cited 2023 Jan 4]. Available from: https://www.r-project.org/.

[34] Little RJA, Rubin DB. Statistical analysis with missing data [Internet]. Hoboken, NJ, USA: John Wiley & Sons, Inc.; 2002. 10.1002/9781119013563.

[35] Malina RM. Tracking of physical activity across the lifespan [Internet]. President’s Council on Physical Fitness and Sports, Washington DC.; 2001 Sep. Available from: https://files.eric.ed.gov/fulltext/ED470692.pdf.

[36] Nader PR, Bradley RH, Houts RM, McRitchie SL, O’Brien M. Moderate-to-vigorous physical activity from ages 9 to 15 years. JAMA [Internet]. 2008;300:295–305. 10.1001/jama.300.3.295. 10.1001/jama.300.3.295 18632544

[37] Steene-Johannessen J, Hansen BH, Dalene KE, Kolle E, Northstone K, Møller NC et al. Variations in accelerometry measured physical activity and sedentary time across Europe - harmonized analyses of 47,497 children and adolescents. Int J Behav Nutr Phys Act [Internet]. 2020;17:38. 10.1186/s12966-020-00930-x. 10.1186/s12966-020-00930-x PMC7079516 32183834

[38] Pedišić Ž, Bauman A. Accelerometer-based measures in physical activity surveillance: current practices and issues. Br J Sports Med [Internet]. 2015;49:219–23. 10.1136/bjsports-2013-093407. 10.1136/bjsports-2013-093407 25370153

[39] Grydeland M, Hansen BH, Ried-Larsen M, Kolle E, Anderssen SA. Comparison of three generations of ActiGraph activity monitors under free-living conditions: do they provide comparable assessments of overall physical activity in 9-year old children? BMC Sports Sci Med Rehabil [Internet]. 2014;6:26. 10.1186/2052-1847-6-26. 10.1186/2052-1847-6-26 PMC4100529 25031839

[40] Ried-Larsen M, Brønd JC, Brage S, Hansen BH, Grydeland M, Andersen LB et al. Mechanical and free living comparisons of four generations of the Actigraph activity monitor. Int J Behav Nutr Phys Act [Internet]. 2012;9:113. 10.1186/1479-5868-9-113. 10.1186/1479-5868-9-113 PMC3463450 22971175

[41] Hansen BHH, Steene-Johannessen J, Kolle E, Udahl K, Kaupang OB, Andersen ID et al. Kartlegging av fysisk aktivitet blant voksne og eldre 2020-22 (Kan3) [Survey of physical activity among adults and the elderly 2020-22 (Kan3)] [Internet]. Folkehelseinstituttet. [cited 2023 Dec 17]. Available from: https://www.fhi.no/publ/2023/kartlegging-av-fysisk-aktivitet-blant-voksne-og-eldre-2020-22-kan3/.

[42] Loe H, Rognmo Ø, Saltin B, Wisløff U. Aerobic capacity reference data in 3816 healthy men and women 20–90 years. PLoS One [Internet]. 2013;8:e64319. 10.1371/journal.pone.0064319. 10.1371/journal.pone.0064319 PMC3654926 23691196

[43] McClain JJ, Abraham TL, Brusseau TA Jr, Tudor-Locke C. Epoch length and accelerometer outputs in children: comparison to direct observation. Med Sci Sports Exerc [Internet]. 2008;40:2080–7. Available from: https://www.ncbi.nlm.nih.gov/pubmed/18981941. 10.1249/MSS.0b013e3181824d98 18981941

[44] Sirard JR, Pate RR. Physical activity assessment in children and adolescents. Sports Med [Internet]. 2001;31:439–54. 10.2165/00007256-200131060-00004. 10.2165/00007256-200131060-00004 11394563

[45] Kristman V, Manno M, Côté P. Loss to follow-up in cohort studies: how much is too much? Eur J Epidemiol [Internet]. 2004;19:751–60. 10.1023/b:ejep.0000036568.02655.f8. 10.1023/b:ejep.0000036568.02655.f8 15469032

