# Supplementary material for: Longitudinal changes in device-measured physical activity from childhood to young adulthood: the PANCS follow-up study
Source: Int J Behav Nutr Phys Act. 2024 Mar 6;21:29. doi: 10.1186/s12966-024-01578-7 (PMC10916240; doi:10.1186/s12966-024-01578-7)
Supplement: Supplementary file 2 — Supplementary Material 2 [file 12966_2024_1578_MOESM2_ESM.docx]

# Comparative analysis between Evenson and Troiano cut-points in young adulthood

This analysis compares using the Evenson cut-points[1] with using the Troiano cut-points[2] in young adulthood, and how this choice affects the results in the paper. The Evenson cut-points are used at ages 9 and 15 years in both analyses. The thresholds for defining different physical activity intensities for both sets of cut-points are shown below:

|  | **Evenson cut-points** | **Troiano cut-points** |
| --- | --- | --- |
| *Intensities* | *Counts per minute* | *Counts per minute* |
| Sedentary | 0-100 | 0-99 |
| Light physical activity | 101-2295 | 100-2019 |
| Moderate physical activity | 2296-4011 | 2020-5998 |
| Vigorous physical activity | >4012 | >5999 |

## Summary of results

*Table 1* displays the estimated marginal means, extracted from the linear mixed models, of time spent (min/day) sedentary, and in light physical activity (LPA), moderate physical activity (MPA), vigorous physical activity (VPA), and moderate-to-vigorous physical activity (MVPA). Time spent sedentary and in LPA remains similar regardless of which cut-points are being used in young adulthood, but a substantial difference occurs in time spent in MPA and VPA – where use of the Troiano cut-points leads to an increase in MPA and decrease in VPA at age 24 mainly due to the higher threshold for classifying movement as VPA with the Troiano cut-points. Smaller changes are seen for MVPA, with somewhat more minutes spent in MVPA with the Troiano cut-points due to a lower threshold for MPA. *Figure 1* visualises this, where a spike in time spent in MPA and a substantial drop in VPA at age 24 years become evident with the Troiano cut-points.

*Figure 2* shows how MVPA changes from age 9 to 24 years within subgroups, with a comparison between the use of the Evenson cut-points and Troiano cut-points at age 24 years. While time spent in MVPA is slightly higher at in general with the use of the Troiano cut-points, the trends and patterns remain similar regardless of choice of cut-points in young adulthood.

The tracking of MVPA and total physical with use of both the Evenson and Troiano cut-points in young adulthood can be found in *Table 2*. In these analyses, both the stability coefficients and odds ratios remain almost identical between the two sets of cut-points.

# Tables

Table 1. Time (min/day) spent in each device-measured intensity at each time point, comparing the Evenson cut-points and Troiano cut-points in young adulthood, respectively. The estimated marginal means are extracted from the linear mixed models, and adjusted for sex, mean wear time of the accelerometer, wear month, a weekday/weekend wear day ratio, and a nested random intercept on an individual level within school clusters at age 9 years.

|  | **Evenson cut-points in young adulthood** | | **Troiano cut-points in young adulthood** | |
| --- | --- | --- | --- | --- |
| Intensities | Estimated marginal means (95% CI) | Change (95% CI) | Estimated marginal means (95% CI) | Change (95% CI) |
| *Sedentary (min/day)* |  |  |  |  |
| Age 9 years | 442.1 (435.2, 448.9) |  | 442.1 (435.3, 449.0) |  |
| Age 15 years | 591.6 (584.9, 598.3) | 149.5 (141.8, 157.3) | 591.6 (584.8, 598.3) | 149.5 (141.7, 157.1) |
| Age 24 years | 581.3 (573.9, 588.8) | −10.3 (−18.5, −2.1) | 581.0 (573.5, 588.4) | −10.6 (−18.8, −2.4) |
| *LPA (min/day)* |  |  |  |  |
| Age 9 years | 286.4 (281.0, 291.9) |  | 286.3 (280.9, 291.7) |  |
| Age 15 years | 161.6 (156.2, 166.9) | −124.8 (130.9, 118.8) | 161.5 (156.2, 166.8) | −124.8 (−130.8, −118.8) |
| Age 24 years | 172.3 (166.4, 178.2) | 10.7 (4.3, 17.2) | 166.7 (160.8, 172.5) | 5.2 (−1.2, 11.6) |
| *MPA (min/day)* |  |  |  |  |
| Age 9 years | 45.8 (44.2, 47.5) |  | 45.5 (43.6, 47.5) |  |
| Age 15 years | 29.6 (27.9, 31.3) | −16.2 (−18.1, −14.3) | 29.2 (27.3, 31.2) | −16.3 (−18.5, −14.0) |
| Age 24 years | 26.0 (24.2, 27.9) | −3.6 (−5.6, −1.5) | 51.2 (49.1, 53.4) | 22.0 (19.6, 24.4) |
| *VPA (min/day)* |  |  |  |  |
| Age 9 years | 30.1 (28.2, 32.7) |  | 30.9 (28.8, 33.0) |  |
| Age 15 years | 22.0 (19.8, 24.2) | −8.5 (−10.8, −6.2) | 22.4 (20.4, 24.5) | −8.5 (−10.5, −6.4) |
| Age 24 years | 25.2 (22.8, 27.6) | 3.2 (0.7, 5.7) | 6.1 (3.8, 8.3) | −16.3 (−18.6, −14.2) |
| *MVPA (min/day)* |  |  |  |  |
| Age 9 years | 76.3 (72.9, 79.7) |  | 76.4 (72.9, 79.8) |  |
| Age 15 years | 51.6 (48.2, 55.0) | −24.7 (−28.3, −21.2) | 51.7 (48.3, 55.1) | −24.7 (−28.3, −21.1) |
| Age 24 years | 51.2 (47.5, 54.9) | −0.4 (−4.2, 3.4) | 57.2 (53.5, 60.9) | 5.5 (1.6, 9.4) |
| LPA: Light physical activity; MPA: Moderate physical activity; VPA: Vigorous physical activity; MVPA: Moderate-to-vigorous physical activity | | | | |

|  | **Crude models** | | | |  | **Adjusted models** | | | |
| --- | --- | --- | --- | --- | --- | --- | --- | --- | --- |
|  | **Evenson cut-points in young adulthood** | | **Troiano cut-points in young adulthood** | |  | **Evenson cut-points** | | **Troiano cut-points** | |
| **Tracking age** | **MVPA** | **Total PA** | **MVPA** | **Total PA** |  | **MVPA** | **Total PA** | **MVPA** | **Total PA** |
| *9-24 (n=640)* | *Estimate (95% CI)* | *Estimate (95% CI)* | *Estimate (95% CI)* | *Estimate (95% CI)* |  | *Estimate (95% CI)* | *Estimate (95% CI)* | *Estimate (95% CI)* | *Estimate (95% CI)* |
| Stability Coefficient | 0.22 (0.14, 0.29) | 0.22 (0.14, 0.29) | 0.22 (0.15, 0.29) | 0.22 (0.14, 0.29) |  | 0.18 (0.09, 0.26) | 0.16 (0.08, 0.24) | 0.18 (0.09, 0.26) | 0.16 (0.08, 0.24) |
| Odds Ratio |  |  |  |  |  |  |  |  |  |
| Lowest Quartile | 1.92 (1.33, 2.76) | 2.10 (1.46, 3.01) | 1.92 (1.34, 2.76) | 2.08 (1.45, 3.00) |  | 1.88 (1.23, 2.86) | 1.87 (1.21, 2.87) | 1.87 (1.22, 2.85) | 1.86 (1.21, 2.86) |
| Highest Quartile | 1.44 (0.99, 2.08) | 1.38 (0.97, 1.97) | 1.40 (0.96, 2.03) | 1.40 (0.98, 2.00) |  | 1.07 (0.69, 1.66) | 1.15 (0.75, 1.76) | 1.02 (0.65, 1.58) | 1.17 (0.76, 1.79) |
| *9-15 (n=458)* |  |  |  |  |  |  |  |  |  |
| Stability Coefficient | 0.29 (0.21, 0.37) | 0.28 (0.20, 0.36) | 0.29 (0.21, 0.37) | 0.28 (0.20, 0.36) |  | 0.24 (0.15, 0.33) | 0.23 (0.13, 0.32) | 0.24 (0.15, 0.33) | 0.23 (0.13, 0.32) |
| Odds Ratio |  |  |  |  |  |  |  |  |  |
| Lowest Quartile | 2.23 (1.47, 3.39) | 2.65 (1.75, 4.03) | 2.23 (1.47, 3.39) | 2.65 (1.75, 4.03) |  | 2.18 (1.34, 3.55) | 2.41 (1.47, 3.96) | 2.18 (1.34, 3.55) | 2.41 (1.47, 3.96) |
| Highest Quartile | 1.83 (1.17, 2.85) | 1.67 (1.10, 2.55) | 1.83 (1.17, 2.85) | 1.67 (1.10, 2.55) |  | 1.23 (0.73, 2.08) | 1.40 (0.85, 2.30) | 1.23 (0.73, 2.08) | 1.40 (0.85, 2.30) |
| *15-24 (n=163)* |  |  |  |  |  |  |  |  |  |
| Stability Coefficient | 0.00 (−0.15, 0.15) | 0.06 (−0.09, 0.21) | −0.01 (−0.16, 0.14) | 0.06 (−0.09, 0.21) |  | −0.05 (−0.22, 0.12) | 0.04 (−0.14, 0.21) | −0.05 (−0.22, 0.12) | 0.04 (−0.14, 0.21) |
| Odds Ratio |  |  |  |  |  |  |  |  |  |
| Lowest Quartile | 1.54 (0.74, 3.22) | 1.72 (0.86, 3.47) | 1.58 (0.76, 3.27) | 1.77 (0.88, 3.57) |  | 1.78 (0.68, 4.68) | 1.92 (0.79, 4.69) | 1.93 (0.74, 5.03) | 2.03 (0.83, 4.97) |
| Highest Quartile | 0.80 (0.39, 1.67) | 1.75 (0.90, 3.41) | 0.80 (0.38, 1.65) | 1.81 (0.93, 3.54) |  | 0.86 (0.34, 2.16) | 1.74 (0.74, 4.08) | 0.84 (0.33, 2.10) | 1.82 (0.77, 4.30) |
| MVPA: Moderate and vigorous physical activity; PA: Physical activity | | | | | | | | | |

Table 2. Tracking of device-measured physical activity from 9 to 24 years of age, comparing the use of Evenson and Troiano cut-points in young adulthood.

# Figures


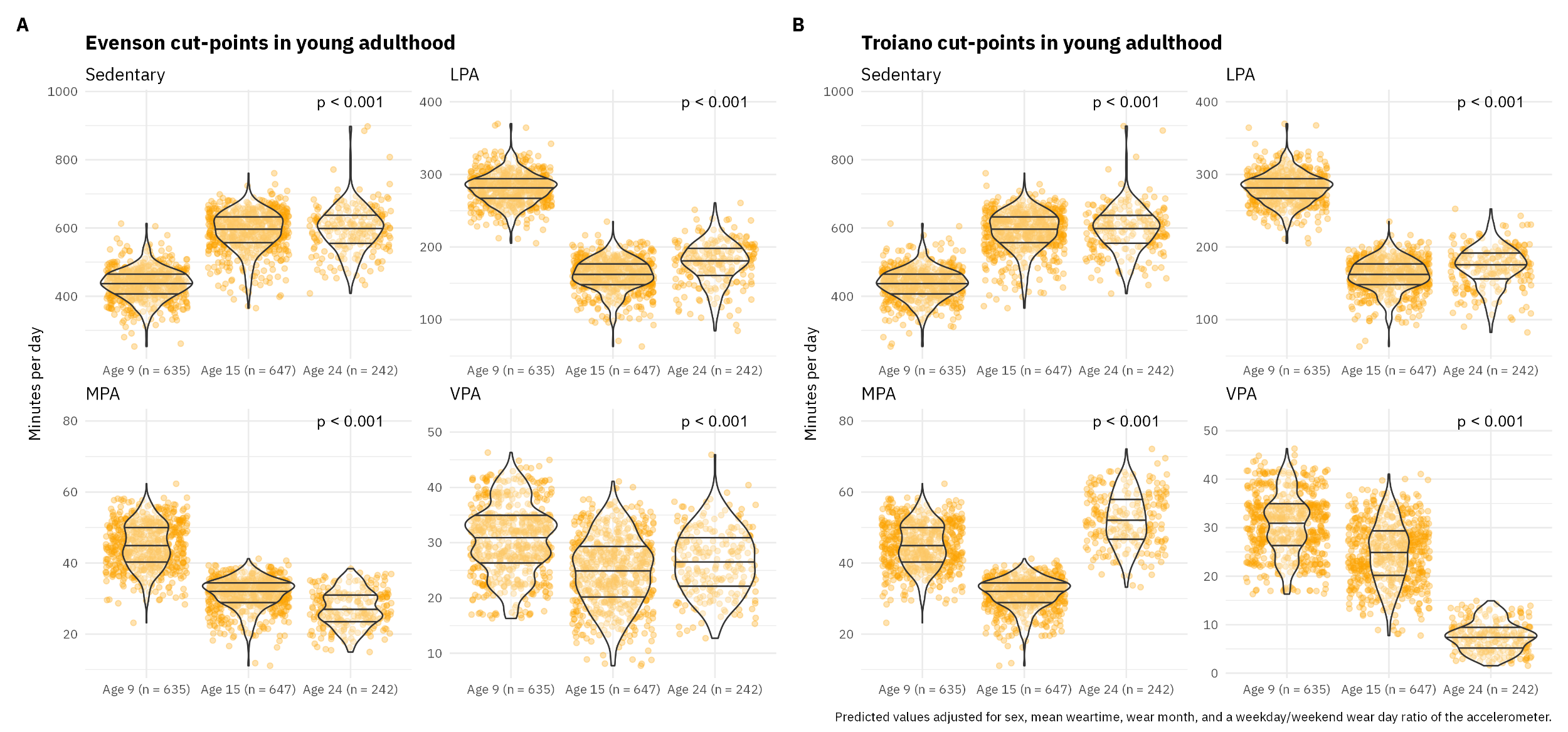


Figure 1. Violin plot of average daily time spent sedentary and in different physical activity intensities from 9 to 24 years of age, where A) displays changes with the use of Evenson cut-points in young adulthood, and B) displays changes with the use of Troiano cut-points in young adulthood. LPA: Light physical activity; MPA: Moderate physical activity; VPA: Vigorous physical activity.


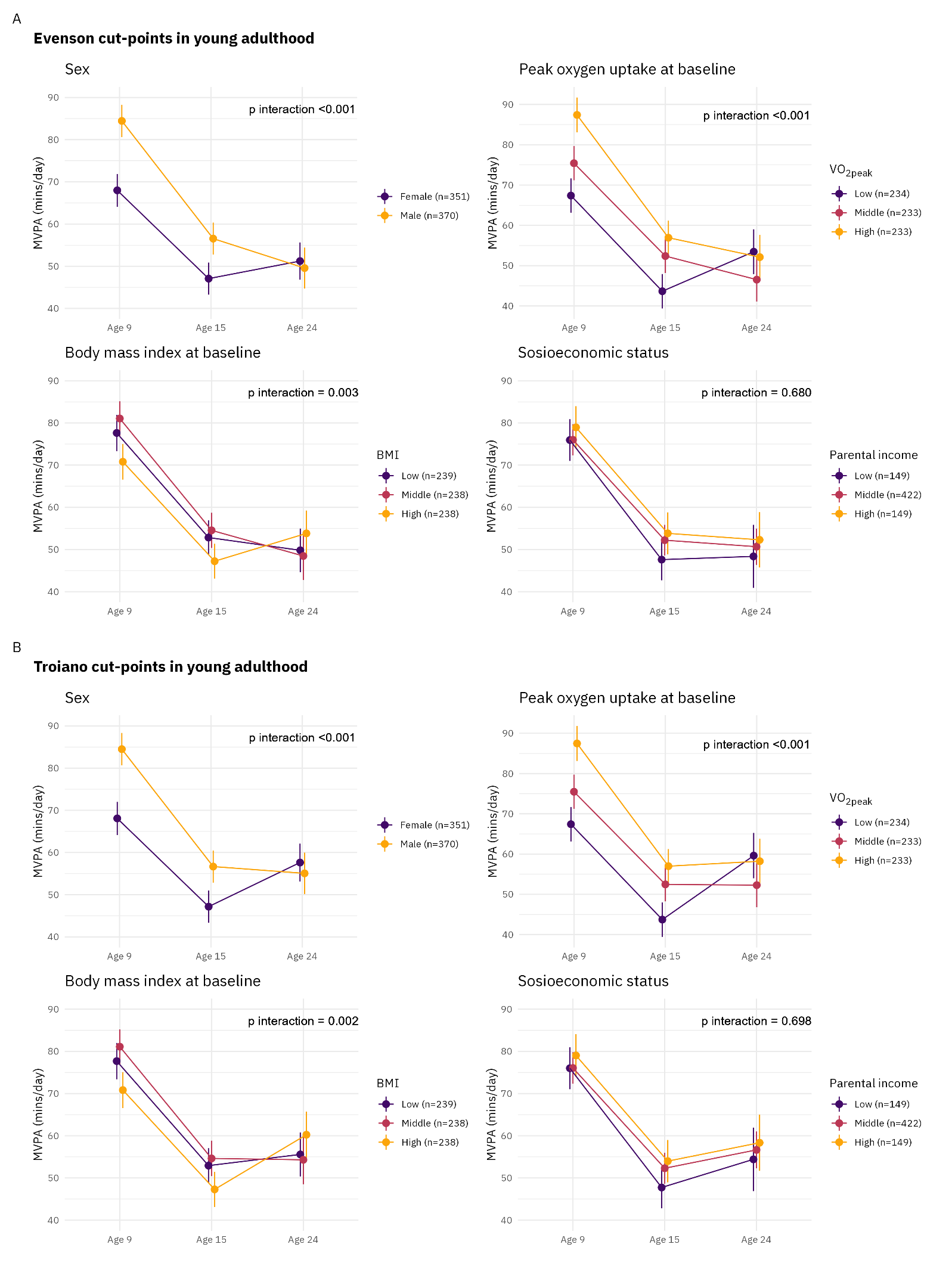


Figure 2. Changes in daily moderate-to-vigorous physical activity (MVPA) from 9 to 24 years, by subgroups of sex, VO_2peak_ at baseline, body mass index at baseline, and parental income. Figure A displays the changes with use of the Evenson cut-points in young adulthood, while the Troiano cut-points are used in young adulthood in Figure B.

# References

1. Evenson KR, Catellier DJ, Gill K, Ondrak KS, McMurray RG. Calibration of two objective measures of physical activity for children. J Sports Sci [Internet]. 2008;26:1557–65. Available from: http://dx.doi.org/10.1080/02640410802334196

2. Troiano RP, Berrigan D, Dodd KW, Mâsse LC, Tilert T, McDowell M. Physical activity in the United States measured by accelerometer. Med Sci Sports Exerc [Internet]. 2008;40:181–8. Available from: http://dx.doi.org/10.1249/mss.0b013e31815a51b3
