# Supplementary material for: Longitudinal changes in device-measured physical activity from childhood to young adulthood: the PANCS follow-up study
Source: Int J Behav Nutr Phys Act. 2024 Mar 6;21:29. doi: 10.1186/s12966-024-01578-7 (PMC10916240; doi:10.1186/s12966-024-01578-7)
Supplement: Supplementary file 4 — Supplementary Material 4 [file 12966_2024_1578_MOESM4_ESM.docx]

# Supplementary Material

**Prediction of physical activity level in young adulthood from collected data at age 9 and 15 years**

Method:

Individual prediction of physical activity level as young adults based on early life factors was analyzed with an extreme gradient boosting model (XGBoost), using a median split of total physical activity level (counts/min) at age 24 years as the outcome variable and all available data at age 9 and 15 years as potential predictors (accelerometer data, anthropometry, physical fitness, blood samples, blood pressure, sexual maturity, socioeconmic status, questionnaires). The analysis was conducted in R v4.2.0 using the *tidymodels* library. Data were randomly divided into training (90%) and testing (10%) data, where model tuning and 10-fold cross-validation were performed on the training data before applying the best model to the remaining test data. Missing data among the predictors were imputed with a combination of random forests and predicted mean matching using the *missRanger* R library. Due to the small number of complete outcome data (n=242), we simulated the extreme gradient boosting model 1000 times to make the model more robust against random variation. The resulting point estimate is the mean across all simulations, while the confidence interval corresponds to the 2.5^th^ and 97.5^th^ percentile, respectively.

Supplementary Table S1: Performance metrics of the extreme gradient boosting model, averaged over 1000 simulations.

| Metric | Estimate (95% CI) |
| --- | --- |
| Accuracy | 0.53 (0.35, 0.69) |
| ROC-AUC | 0.53 (0.33, 0.71) |

Supplementary Table S2: The top 5 variables contributing to prediction improvement, by gain in accuracy from the predictor.

| Variable | Importance (average gain) |
| --- | --- |
| Total PA at age 15 | 0.22 |
| Mean MVPA at age 15 | 0.21 |
| Mean VPA at age 15 | 0.12 |
| Serum adiponectin at age 9 | 0.09 |
| Mean MPA at age 9 | 0.08 |

**Comparison between completers and drop-outs**

Supplementary Table S3: Comparison of completers of all three studies with participants who dropped out.

|  | **Age 9** | | | **Age 15** | | | | |
| --- | --- | --- | --- | --- | --- | --- | --- | --- |
| *Variable* | *Missing* | *Completer*, n = 258*^1^* | *Dropout*, n = 473*^1^* | | *Missing* | *Completer*, n = 258*^1^* | *Dropout*, n = 473*^1^* |  |
| Age (yrs) | 0% | 9.6 (0.4) | 9.6 (0.4) | | 0% | 15.3 (0.6) | 15.1 (0.6) |  |
| Height (cm) | 0.8% | 138.7 (6.1) | 139.2 (6.7) | | 9.3% | 169.2 (8.5) | 170.0 (8.4) |  |
| Weight (kg) | 0.8% | 32.7 (5.7) | 33.8 (6.9) | | 11% | 58.2 (9.9) | 60.7 (11.0) |  |
| Waist Circumference (cm) | 1.0% | 61.5 (6.2) | 62.1 (7.6) | | 17% | 69.9 (6.5) | 71.4 (8.0) |  |
| BMI (kg/m^2^) | 0.8% | 16.9 (2.1) | 17.4 (2.6) | | 11% | 20.3 (2.6) | 21.0 (3.2) |  |
| VO_2peak_ (ml/kg/min) | 2.9% | 46.8 (6.8) | 46.2 (7.8) | | NA | NA | NA |  |
| Sex | 0% |  |  | | 0% |  |  |  |
| Female |  | 141 (55%) | 213 (45%) | |  | 141 (55%) | 213 (45%) |  |
| Male |  | 117 (45%) | 260 (55%) | |  | 117 (45%) | 260 (55%) |  |
| Pubertal status (2005-2006) | 1.5% |  |  | | 1.5% |  |  |  |
| Pre-pubertal |  | 224 (87%) | 391 (85%) | |  | 224 (87%) | 391 (85%) |  |
| Started puberty |  | 34 (13%) | 71 (15%) | |  | 34 (13%) | 71 (15%) |  |
| Region (2005-2006) | 16% |  |  | | 16% |  |  |  |
| Central Norway |  | 7 (3%) | 20 (5%) | |  | 7 (3%) | 20 (5%) |  |
| East |  | 137 (62%) | 180 (47%) | |  | 137 (62%) | 180 (47%) |  |
| North |  | 23 (10%) | 40 (10%) | |  | 23 (10%) | 40 (10%) |  |
| Southwest |  | 55 (25%) | 150 (38%) | |  | 55 (25%) | 150 (38%) |  |
| Parental income (2011-2012) | 0.1% |  |  | | 0.1% |  |  |  |
| Low |  | 44 (17%) | 107 (23%) | |  | 44 (17%) | 107 (23%) |  |
| Middle |  | 153 (59%) | 275 (58%) | |  | 153 (59%) | 275 (58%) |  |
| High |  | 61 (24%) | 90 (19%) | |  | 61 (24%) | 90 (19%) |  |
| Norwegian parents | 0% |  |  | | 0% |  |  |  |
| None |  | 17 (6.6%) | 50 (11%) | |  | 17 (6.6%) | 50 (11%) |  |
| One |  | 43 (17%) | 94 (20%) | |  | 43 (17%) | 94 (20%) |  |
| Two |  | 198 (77%) | 329 (70%) | |  | 198 (77%) | 329 (70%) |  |
| *Accelerometer data* |  |  |  | |  |  |  |  |
| Valid Weardays | 13% | 3.7 (0.6) | 3.7 (0.6) | | 11% | 6.7 (1.8) | 6.2 (1.9) |  |
| Wear time (hrs/day) | 13% | 13.3 (1.0) | 13.1 (1.2) | | 11% | 13.7 (1.2) | 13.3 (1.4) |  |
| Sedentary (min/day) | 13% | 439 (393, 476) | 433 (383, 478) | | 11% | 603 (560, 651) | 589 (532, 636) |  |
| LPA (min/day) | 13% | 279 (251, 313) | 279 (248, 317) | | 11% | 162 (139, 188) | 158 (132, 189) |  |
| MPA (min/day) | 13% | 43 (36, 53) | 44 (35, 53) | | 11% | 32 (25, 40) | 29 (22, 37) |  |
| VPA (min/day) | 13% | 30 (22, 38) | 27 (19, 37) | | 11% | 26 (16, 35) | 20 (13, 31) |  |
| MVPA (min/day) | 13% | 73 (59, 90) | 72 (57, 88) | | 11% | 58 (44, 74) | 50 (38, 68) |  |
| Total PA (counts/min) | 13% | 682 (572, 848) | 661 (551, 827) | | 11% | 433 (354, 549) | 408 (331, 519) |  |
| *^1^*Mean (SD); n (%); Median (IQR)  NA: Not available; BMI: Body mass index; VO_2peak_: Peak oxygen uptake; LPA: Light physical activity; MPA: Moderate physical activity; VPA: Vigorous physical activity | | | | | | | | |
| Note: Completers defined as having participated in all three studies, otherwise dropout. | | | | | | | | |
