# Supplementary material for: Longitudinal changes in device-measured physical activity from childhood to young adulthood: the PANCS follow-up study
Source: Int J Behav Nutr Phys Act. 2024 Mar 6;21:29. doi: 10.1186/s12966-024-01578-7 (PMC10916240; doi:10.1186/s12966-024-01578-7)
Supplement: Supplementary file 3 — Supplementary Material 3 [file 12966_2024_1578_MOESM3_ESM.docx]

Anders Husøy, MSc
Department of Sports Medicine

Norwegian School of Sport Sciences
Pb 4014 Ullevål Stadion, 0806 Oslo, Norway

[andershu@nih.no](mailto:andershu@nih.no)

Prof. Richard Rosenkranz, PhD

Prof. Melanie Hingle, PhD

Editor-in-Chiefs

*International Journal of Behavioral Nutrition and Physical Activity*

22-Dec-2023

Dear Editorial Team and Reviewers,

Thank you to the Editorial Team for considering this manuscript and to the Reviewers for taking the time to read and comment on the paper. A detailed response to each comment, as bulleted points in italic text, can be found on the following pages along with an indication of where in the manuscript changes have been made. The changes are further highlighted in the revised manuscript.

For any further questions, do not hesitate to contact me at [andershu@nih.no](mailto:andershu@nih.no).

Sincerely,

Anders Husøy, MSc

PhD candidate, Department of Sports Medicine

Norwegian School of Sport Sciences

**Comments to the author (if any):**

**Reviewer #1: General comments:**

Thank you for the opportunity for reviewing this manuscript. This study aims to assess changes in device-measured physical activity and sedentary time from childhood through adolescence into young adulthood in Norwegian individuals participating in the PANCS project. Overall, the manuscript is well written, and only minor suggestions are included in this review.

Minor comments:

[Title/Keywords]

- If possible, please include the term 'longitudinal' in the title or keywords as this term is highlighted throughout the manuscript.

- *The term ‘longitudinal’ has been included in the title.*

[Abstract]

- Please revise the background section to highlight research gaps and include the aim of this study.

- *The background section has been revised (lines 24-28).*

- "A total of 731 Norwegian boys and girls… at age 9 years (2005-2006) and 15 years (2011-2012), whereas 258 of these participated again at age 24 years (2019-2021)." - This sentence is confusing after reading the whole manuscript. It sounds that the number of samples are the same in PANCS 1 and 2, but different in the follow-up. The term 'whereas' may not be appropriate here because it is not really a comparison.

- *Figure 1 may be misleading here, as the PANCS studies consists of a cross-sectional arm and a longitudinal arm which were intertwined in PANCS1&2. The longitudinal sample in PANCS consists of 731 boys and girls participating at age 9 and 15 years, and 708 of these were eligible to participate again in the follow-up study at age 24 years. We have modified Figure 1 to better reflect this.*

- In addition, the follow-up study was conducted during the covid-19 pandemic (as the authors described in the discussion), and this event can be mentioned in the abstract given the generalisability of the findings (e.g. "…at the age of 24 years (2019-2021; including the COVID-19 pandemic period)").

- *The mention of the COVID-19 pandemic has been included in the abstract (line 31).*

- It is not very clear about the use of tilde symbols (~) in the results (also in the main text on pages 10-11).

'BMI' and 'VO2peak' should be spelt out at their first mention.

- *The tilde symbols were intended in the mathematical sense, in front of a single quantity, to mean ‘approximately’ due to rounding of the numbers. However, they are not necessary and have been removed throughout (lines 37-38; lines 206-210). The abbreviations BMI and VO_2peak_ have been spelt out in the abstract (line 41).*

[Introduction]

- In the first two paragraphs, the evidence was purely focuses on physical activity but sedentary behaviour. However, sedentary time was suddenly mentioned in the third paragraph. Please provide some evidence on sedentary behaviour in the first two paragraphs.

- *Both physical activity and sedentary time have now been included in the first paragraph (lines 55-60).*

[Material and methods]

The study sample section may need revision to clarify a few points below:

- Please use the term 'wave' (or something similar, e.g. arm) to explain the first wave of PANCS (i.e. PANCS1). The PANCS1 was sometimes referred to 'the first study' in the manuscript which is a little bit confusing.

- *We understand how this is confusing. The PANCS studies were initially intended as a series of cross-sectional studies to monitor secular trends in physical activity among Norwegian children and adolescents. However, an opportunity arose in PANCS2 to create a longitudinal arm of PANCS where all participants aged 9 years in PANCS1 (2005-2006) were invited to participate in PANCS2 as 15-year-olds (2011-2012). Hopefully the modified Figure 1 helps to explain the structure of the different PANCS studies. Furthermore, terms such as ‘the first study’ have been substituted with more specific terms, e.g., PANCS1, throughout.*

- "In the first Physical Activity… to create a nationally representative sample of 9- and 15-year-olds." What happened to the 15-year-old cohort in PANCS1? They were not recruited in PANCS2? The following sentence also says, "then aged 14-16 years" - what does it mean?

- *The 15-year-olds in PANCS1 were only part of the cross-sectional study. The line ‘then aged 14-16 years’ means that those who were 9 years old in PANCS1 were aged 14-16 years at the time of PANCS2 (average age 15 years), and these were invited to participate in PANCS2 to create a longitudinal cohort. This sentence has been changed to clarify that they were 15 years at the time of PANCS2 (line 83).*

- "This constituted a longitudinal cohort of 9- and 15-year-olds distributed across 40 schools…" So the 15-year-old cohort is still part of PANCS2? How many schools were approached in PANCS1?

- *The 15-year-olds in the longitudinal arm of PANCS, at the time of PANCS2, were recruited from the pool of participants who participated in PANCS1 as 9-year-olds. Statistics Norway selected the schools eligible for invitation based on population density, geography, and socioeconomic status to create a nationally representative sample. If schools declined to participate, Statistics Norway selected new schools based on the same criteria until the sample size reached a pre-specified number. In total, participants were recruited from 40 elementary schools and 23 high schools in PANCS1.*

- "In 2019, those who participated in both PANCS1 and PANCS2… for inclusion in the PANCS follow-up study." Please briefly describe how the participants recruited in the follow-up wave, and any adjustment to recruitment made during the COVID-19 pandemic.

- *The recruitment process in the follow-up wave, and adjustments due to the COVID-19 pandemic have been added to this section (lines 86-92).*

- Please confirm that Statistics Norway only provided information on parental education level, income and country of birth at the time of PANCS2, but PANCS1. The socio-demographic data in PANCS 1 (at the age of 9) are listed in Table 1 and Supplementary Table S3. Does it mean that the same individual data were used across PANCS1, 2 and follow-up in these variables?

- *Yes, these variables were only provided from Statistics Norway in PANCS2 (2011-12). While country of birth would not change, parental income may vary across the life span. However, as we only had access to the variables as they were in 2011-12, they were treated as fixed variables. Table 1 and Supplementary Table S3 have been updated to inform when these variables were collected.*

- "Data between 00:00 and 06:00 were excluded at age 9 and 15, but not at age 24 due to larger uncertainty surrounding sleep habits and known nightshift work for several of the participants." How did this decision impact on the total amount of sedentary time in the follow-up datasets (at the age of 24)? If sleeping time was not taken out from the data in the follow-up wave, it is likely that sedentary time is longer (proportionally).

- *Sleeping time was removed from the data at all points through the non-wear algorithm, as the participants were instructed to remove the accelerometer for sleep and water-based activities. Removal of data between 00:00 and 06:00 was done to remove any potential noise in data under the assumption that 9- and 15-year-olds would be asleep at these hours. However, inspection of the wear-time post-processing showed that the non-wear algorithm did a very good of removing sleep time – and thus removing data between 00:00 and 06:00 at age 9 and 15 years may not have been needed as these data were removed through the non-wear algorithm. Furthermore, time spent in different intensities are included in Table 1 and shows that both wear time and sedentary time were similar at ages 15 and 24 years.*

- Please include descriptive statics of other covariates (geographical area, pubertal status, ethnicity) in Table 1. Please include the description of ethnicity in the other covariates section too.

- *These variables have been added to Table 1. The term ‘ethnicity’ may be misleading as it refers to the parents’ country of birth, categorised as the number of Norwegian parents. Accordingly, the term ‘ethnicity’ has been removed throughout.*

- In the statistical analyses section, please specify which wave of 'wear time', 'wear month', and 'weekday/weekend war day ratio' were used in the models. As described above, was the parental income variable from PANCS 2 used in the analysis?

- *The data were analysed in ‘long’ format, structured hierarchically on an individual level. Thus, the accelerometer measurements at each time point were adjusted for the corresponding values of wear time, wear month, and weekday/weekend wear day ratio. The parental income variable, collected in PANCS2, were included in the adjusted tracking models but not in the analysis of change in physical activity and sedentary time (adjusted for sex, wear time, wear month, and the weekday/weekend wear day ratio).*

- There is a typo - it should be 'weekday/weekend ratio' rather than 'weekday/weekday ratio' (between lines 40-41).

- *Thank you, this has been corrected (line 172 in the revised manuscript).*

- Interpretation of 'stability coefficient*' (i.e. *weak tracking: <0.30, moderate tracking: 0.30-0.60, strong tracking: >0.60) can be included in the Table 2 as a footnote.

- *This has been included in Table 2 as a footnote.*

[Results]

- Figure 1 and the first paragraph are confusing:

"Participation rate 89%" - Does it mean 89% of 1306, or 1306 was 89% of 1467 (originally recruited for PANCS1)?

- *This may be confusing due to the fact that the PANCS studies have both a cross-sectional and longitudinal arm. We have changed the first paragraph in the results to be more precise (lines 186-193).*

- What does "who were found" mean?

- *It was intended to mean those who were identified, but this has been removed and the paragraph rewritten as replied above.*

- In Figure 1, what do numbers for 'invited' and 'responses' indicate? Are they different participants (what do 'invited' and 'responses' mean)? As the side arrows drawing between PANCS1 and 2 as well as PANCS2 and follow-up, the numbers should be equivalent to 575 (1306-731) and 473 (731-258), respectively? Please clarify the numbers in Figure 1.

- *Figure 1 have been changed to clarify the structure of the PANCS studies, which have both a cross-sectional and longitudinal arm.*

- Based on Figure 1 and Table 1, more than half of the participants in PANCS1 (671 out of 1306) did not have valid accelerometer data. From the author's point of view, was this also potentially due the storage limitation of the accelerometers?

- *The number of participants was 731 at age 9 and 15 years, which means that the number of valid accelerometer data was 635 out of 731 at age 9 years and 647 out of 731 at age 15 years. The most likely sources of non-valid measurements were not wearing the accelerometer and device errors.*

- Results from the interactions terms are very interesting, and how they were compared with the machine learning algorithms in the discussion!

- *Thank you, we thought so as well.*

[Discussion]

- The discussion section is very thorough in general. There is one suggestion. Please provide some context of Norwegian physical activity in general - how this PANCS cohort can be representative. Also the physical activity level measured at the age of 24 was comparable to the previous national data (if available) - there is little concerned that the level of physical activity may be lower and sedentary behaviour higher in the follow-up due to the covid-19 pandemic (an issue of generalisability).

- *We have added some context of how the PANCS follow-up cohort can be representative in the discussion (lines 302-306). While one may have expected the pandemic to affect physical activity (which it probably did in terms of activity types and arenas), there was no indication of lower physical activity level in the follow-up due to the COVID-19 pandemic.*

**Reviewer #2: General comments:**

This is an interesting and well-written manuscript examining age-related changes in physical activity and the tracking of these behaviours. It is great to see a longitudinal study using device-based approaches to monitor activity from childhood into young adulthood. The results are highly relevant for researchers and policy makers/practitioners. I have outlined some specific comments below, mainly related to the accelerometer processing.

Specific comments:

Abstract: Summarises the key information and results well.

Introduction:

- Lines 27-30 - it would be good to elaborate on the existing tracking evidence, including the strength of identified tracking coefficients.

- *Existing tracking evidence and the strength of tracking coefficients have been added to the introduction (lines 66-68).*

- Concise, clear introduction.

Methods:

- Several different monitors were used, how have the study team accounted for the variability between the different models?

- *The accelerometer models used at age 15 and 24 years are comparable but the CSA7164 used at age 9 years records somewhat less sedentary time and higher total activity than the newer models. Some have suggested to apply a correction factor to mean physical activity (cpm) to attenuate the differences*[1]*, while others have warned that this may cause a bias of unknown magnitude due to the inter-generation differences varying across intensities*[2]*. Another option is to enable a low frequency extension option in the ActiLife software. However, while this attenuates the differences between the monitors for time spent sedentary and in light physical activity, it introduces a bias for time spent in moderate physical activity*[3]*. Therefore, no steps were taken to account for this – other than discussing the differences between the models. Furthermore, the observed absolute differences between these models are relatively modest*[2]*, and much smaller than the changes we observed between 9 and 15 years. Thus, we do not believe that using the same accelerometer across all three time points would have made any substantial difference to the results.*

- For the follow up, presumably because sleep time was not accounted for sedentary estimates were not used in analysis? Also what impact does this have on wear time?

- *With correct usage of the monitors, sleep time would be classified as non-wear as the participants were instructed at all time points to remove the monitor for sleep and water-based activities. Data between 00:00 and 06:00 were filtered out to remove any potential noise at age 9 and 15 years under the assumption that youth at these ages would normally sleep at these hours. We did not filter away data at these hours in young adulthood, as sleep patterns may vary substantially among adults and we were informed by several of the participants that they worked night shifts. Indeed, by inspection of the raw wear files we saw that the monitors very rarely recorded movement after midnight at the ages of 9 and 15 years. However, it was not uncommon to see recorded movement after midnight at age 24 years, especially during weekend days. On occasions of late-night movement, a corresponding late start time of wearing the monitor the next day was usually seen. Thus, there was no impact from sleep on the wear time, and average wear time of the monitors was similar at age 15 (13.5 hrs/day) and 24 years (13.8 hrs/day).*

- Justification needed for the 8hrs, 2days inclusion criteria.

- *Justification for these criteria has been included in the methods chapter (lines 123-126).*

- The thresholds for the follow up phases when the participants were adults are not appropriate to use as they are calibrated and validated for use in children. This will likely lead to an underestimation in adult's MVPA. Have the authors considered the impact of this? I would urge the authors to re-analyse the follow up data using age-appropriate thresholds.

- *There is a plethora of different accelerometer cut-points, and choice of cut-points can substantially change the estimates of sedentary time, physical activity, and whether the recommendations for physical activity are met* [4–6] *– even if they are validated for use in the same population. While there is no optimal solution to the selection of cut-points in a longitudinal study from childhood to young adulthood, we opted to implement the same cut-points in all three study waves, as changing the cut-points would pose the question as to whether it was the physical activity level or the different sets of cut-points responsible for any observed change. However, the physical activity data at age 24 were also analysed with the Troiano cut-points validated in adults* [7]*, and a comparative analysis between the use of the Evenson and Troiano cut-points in young adulthood is attached to this revision. In short, time spent in moderate and vigorous physical activity changes due to different thresholds but the conclusions for the interaction and tracking analyses remain the same regardless of which cut-points are being used at age 24 years. Furthermore, we included the measure of total physical activity (counts/min) in Table 1, which is independent of these thresholds. This shows a substantial reduction from age 9 to 15 years but only a minor decrease from age 15 to 24 years, which is more in line with the results from the Evenson cut-points than the Troiano cut-points in young adulthood.*

- Analysis- did the team examine whether data were missing at random?

- *While we cannot test whether data is missing at random or missing not at random, as the latter depends on unobserved data, we did a drop-out analysis as shown in Additional file 1 (Supplementary Table S3) – revealing some minor differences in waist circumference, sex, geographical region, socioeconomic status, number of Norwegian parents, and accelerometer wear. The large number of variables in our dataset is supportive of the MAR assumption (missing data can be explained by the observed data), and we are confident most of the missing data are explained by the observed data.*

- Is it appropriate to examine sedentary time for the participants in the follow up phase as sleep was not subtracted and as a result sedentary estimates would be substantially higher? I know this would be adjusted for wear time, but it may warrant further consideration.

- *See answer above regarding sleep time. With per protocol usage of the accelerometer, i.e., removing the monitor for sleep and water-based activities, sleep time would be classified as non-wear and would not influence the estimates of sedentary time.*

Results:

- As mentioned earlier, the follow-up data would benefit from being re-processed using cutpoints that are validated in adults. That may change the MVPA estimates substantially.

- The age related changes between 9-15 are as expected, the changes between 15-24 may change if different cutpoints are used.

- Very interesting tracking data, again with the caveat about the accelerometer processing for the follow-up data.

- *The change of cut-points influenced time spent in moderate physical activity (increased) and time spent in vigorous physical activity (decreased) due to different thresholds – see the comparative analysis for details. However, the interaction and tracking analyses remained similar regardless of choice of cut-points at age 24 years.*

Discussion:

- The discussion covers the key areas well- but may change if authors complete additional analysis.

- In the limitations, some steps of the accelerometer processing should be covered. For example the use of 10s epochs, which likely resulted in an underestimation of VPA in the 9yr olds. Also the non-removal of sleep time at follow up, and the inclusion criteria.

- *The use of 10s epochs is now included in the limitations (lines 307-311). See above regarding sleep time. A short comparison of the cohort at age 24 years to other studies of Norwegian young adults has also been included in the discussion (lines 302-306).*

# References

1.	Corder K, Brage S, Ramachandran A, et al (2007) Comparison of two Actigraph models for assessing free-living physical activity in Indian adolescents. J Sports Sci 25:1607–1611. https://doi.org/10.1080/02640410701283841

2.	Grydeland M, Hansen BH, Ried-Larsen M, et al (2014) Comparison of three generations of ActiGraph activity monitors under free-living conditions: do they provide comparable assessments of overall physical activity in 9-year old children? BMC Sports Sci Med Rehabil 6:26. https://doi.org/10.1186/2052-1847-6-26

3.	Ried-Larsen M, Brønd JC, Brage S, et al (2012) Mechanical and free living comparisons of four generations of the Actigraph activity monitor. Int J Behav Nutr Phys Act 9:113. https://doi.org/10.1186/1479-5868-9-113

4.	Watson KB, Carlson SA, Carroll DD, Fulton JE (2014) Comparison of accelerometer cut points to estimate physical activity in US adults. J Sports Sci 32:660–669. https://doi.org/10.1080/02640414.2013.847278

5.	Trost SG, Loprinzi PD, Moore R, Pfeiffer KA (2011) Comparison of accelerometer cut points for predicting activity intensity in youth. Med Sci Sports Exerc 43:1360–1368. https://doi.org/10.1249/MSS.0b013e318206476e

6.	Pedišić Ž, Bauman A (2015) Accelerometer-based measures in physical activity surveillance: current practices and issues. Br J Sports Med 49:219–223. https://doi.org/10.1136/bjsports-2013-093407

7.	Troiano RP, Berrigan D, Dodd KW, et al (2008) Physical activity in the United States measured by accelerometer. Med Sci Sports Exerc 40:181–188. https://doi.org/10.1249/mss.0b013e31815a51b3
